# Influence of Circadian Rhythm on the Surgical Stress Response in Bitches Undergoing Elective Ovariohysterectomy

**DOI:** 10.3390/ani16050795

**Published:** 2026-03-04

**Authors:** Pauline Silva dos Santos, Luísa Pereira Zacchi, Maria Helena Moreno, Márcio Oleszczyszyn, Heloísa Vieira Cordeiro, Lincoln Gonçalves Marcilio, Dalila Moter Benvegnú, Felipe Beijamini, Camila Dalmolin, Tatiana Champion, Gentil Ferreira Gonçalves, Fabíola Dalmolin

**Affiliations:** 1 Program of Post Graduation in Saúde, Bem-Estar e Produção Animal Sustentável na Fronteira Sul (PPG-SBPAS), Universidade Federal da Fronteira Sul (UFFS), Realeza 85770-000, Brazil; pauline.santos@estudante.uffs.edu.br (P.S.d.S.);; 2 Course of Veterinary Medicine, Universidade Federal da Fronteira Sul (UFFS), Realeza 85770-000, Brazil; 3 Course of Biological Sciences, Universidade Federal da Fronteira Sul (UFFS), Realeza 85770-000, Brazil; 4 Course of Veterinary Medicine, Centro Universitário Mater Dei (UNIMATER), Pato Branco 85501-200, Brazil

**Keywords:** cortisol, leukogram, oxidative stress, physical parameters, inflammatory response

## Abstract

Circadian rhythm regulates several physiological functions, and influences endocrine and metabolic responses in mammals, with cortisol acting as important modulator of this mechanism. Cortisol secretion is affected by both internal and external factors and is intensified under stress conditions. The response to surgical stress is consistently observed after surgical procedures, such as ovariohysterectomy (OVH). Therefore, this study aimed to evaluate the influence of the circadian rhythm on the surgical stress response following elective OVH in healthy bitches. Twenty patients weighing between 10 and 20 kg were hospitalized 48 h before surgery and remained hospitalized for 48 h postoperative. The animals were randomly allocated into two groups and underwent OVH either in the morning (6–8 h—a.m., GAM) or at night (18–20 h—p.m., GPM). Surgical procedures were standardized with respect to the surgical team, technique applied and duration; this was carried out in order to induce a comparable level of surgical stress. Physical parameters (systolic blood pressure, heart rate, respiratory rate and rectal temperature) and laboratorial analyses (cortisol, leukogram, protein thiols, no protein thiols, vitamin C, ferric reducing ability of plasma and thiobarbituric acid reactive substances) were assessed immediately before surgery and at 2, 4, 6, 12, 24 and 48 h, as well as 14 days postoperatively. No significant changes in cortisol profile were detected. However, significant alteration in the respiratory rate, rectal temperature, time to first urine, and lipid peroxidation were observed in the GPM group, suggesting that surgeries performed at night induce greater disturbances in homeostasis than those performed in the morning.

## Simple Summary

This study evaluated the influence of the circadian rhythm on the postoperative recovery of bitches undergoing elective ovariohysterectomy (OVH). Clinical and laboratorial assessments were performed after surgeries conducted at different times of the day (morning or night). The data suggest that procedures performed in the evening or at night induce greater disturbances in homeostasis than those performed in the morning. The response to surgical stress may impair postoperative recovery and is affected by anesthetic management, surgical trauma, duration, and the patient’s condition. Understanding the mechanisms that modulate postoperative physiological alterations, is essential to minimize complications associated with an exacerbated surgical stress response.

## 1. Introduction

Ovariohysterectomy (OVH) is one of the most common surgical procedures performed in small animals [1]. It is indicated both for population control and for treatment of reproductive diseases. The technique is well established, allowing procedural standardization and predictability of the surgical time [1]. Furthermore, it is performed appropriately by a trained surgical team [2]. The physiological response to surgical stress induced by this procedure is also well described in the literature [3,4,5,6,7,8], which supports its use as an experimental model.

Surgical stress is a consistently observed after procedures and results in a physiological response that includes inflammatory, endocrine, metabolic and immunological alterations [3,4]. This mechanism is essential for adaptation, however, if exacerbated, it may compromise recovery and increase susceptibility to infection [5]. The central nervous system identifies potentially harmful stimuli, and activates compensatory mechanisms to restore the homeostasis, including activation of the hypothalamic–pituitary–adrenal (HPA) axis and the sympathetic nervous system, leading to increased secretion adrenocorticotropic hormone and cortisol [4,6].

There are no specific biomarkers capable of precisely quantifying the stress and pain associated with surgical interventions [4,7]. Therefore, investigations should include an integrated assessment combining physiological parameters and biochemical markers [4,7]. Nociceptive transmission to the brainstem, thalamus and hypothalamus stimulates cardiovascular and respiratory centers, resulting in peripheral vasoconstriction, tachycardia, and increasing myocardial contractility, systemic vascular resistance, oxygen demand, and oxygen consumption [4].

Biomarkers, such as cortisol, are widely used to evaluate the response to surgical stress in veterinary patients particularly dogs [4,8]. Plasma cortisol concentration is modulated by the circadian rhythm; however, external factors may also activate the HPA axis. Nevertheless, stress is the primary cause of increased cortisol concentrations, representing a biological response aimed at restoring homeostasis [9,10].

Following stimulus, such as surgery, the immune system may be either activated or suppressed, depending on the nature and intensity of the stressor. This response involves immunological mediators, including cytokines or interleukins low-molecular-weight proteins produced by leucocytes, fibroblasts and endothelial cells [4]. The leukogram is an important biomarker that, when interpreted alongside other parameters, assists in evaluating the stress response associated with surgical procedures. Corticosteroids also influence the immune response. The so-called stress leukogram in dogs, is characterized by leukocytosis, neutrophilia, lymphopenia, monocytosis, and eosinopenia. This pattern typically develops 4–8 h after an injury and may resolve within 24 h or persist for several days, depending on the duration of corticosteroid exposure [11].

Oxidative stress is another relevant marker for surgical stress and occurs when there is an imbalance between reactive oxygen species (ROS) and antioxidants defenses [12]. In excess, ROS can cause cellular damage in organs, such as the kidney and liver, thereby delaying tissue healing [13]. Although ROS react with various biomolecules, their primary targets include unsaturated lipids and intracellular thiols. Oxidative stress leads to irreversible oxidation of protein thiols, depletion of reduced glutathione (GHS) and potential cell death [14]. Antioxidants defenses include both enzymatic and no enzymatic components. Within the enzymatic system, superoxide dismutase, glutathione peroxidase and catalase, play key roles in neutralizing ROS and protecting cells from oxidative damage [15]. Non enzymatic antioxidants include vitamin C, which plays a fundamental role in cellular defense [15]. The ferric reducing ability of plasma (FRAP) is an indirect method used to evaluate antioxidant status, by determining the ability of plasma to reduce a ferric complex, thereby indicating total antioxidant capacity (TAC) [16]. Malondialdehyde (MDA) is commonly used as an indicator of lipid peroxidation [5], and is indirectly measured by the thiobarbituric acid reactive substances (TBARS) [17].

Circadian rhythm regulates physiological functions in approximately 24 h cycles and modulates several processes, including endocrine and metabolic response in mammals [18]. Cortisol secretion is influenced by this system as well as internal and external factors and is further intensified during stress via activation of the HPA axis [9,10]. Given that plasma cortisol concentration typically peak in the early morning and decline throughout the day, it can be hypothesized that surgical stress response may vary according to the time of the day at which the procedure is performed. Circadian regulation of leukocyte variation throughout the day has been described in dogs [19]. In humans undergoing hip surgery, procedures performed in the afternoon were associated with a faster return of cortisol levels to baseline compared with morning surgeries, and low levels of inflammatory cytokines were observed 1–2 days postoperatively in this group [20].

Therefore, this study evaluated the surgical stress response in bitches undergoing elective OVH performed at different times of the day (morning and night), through clinical parameters, leukogram, cortisol levels and oxidative metabolism markers. Understanding this interaction may clarify the influence of the circadian rhythm on postoperative recovery in these patients.

## 2. Materials and Methods

### 2.1. Animal Selection and Pre Operative Procedures

The study was approved by the institutional Animal Experimentation Ethics Committee of Universidade Federal da Fronteira Sul, Brazil (Protocol No. 893.322.11-23 CEUA) and was conducted in the city of Realeza (25°46′ S, 53°31′ W), during autumn/winter. Owners were informed about surgical and anesthetic risks. Twenty healthy bitches aged between one and four years, weighing 10–20 kg, with a body score of 5–6/9, and up to date on deworming and vaccination were selected. Brachycephalic breeds were excluded.

Health status was confirmed by the absence of clinical disease, and all animals underwent a comprehensive pre-anesthetic evaluation. Detailed physical examinations, abdominal ultrasonography and electrocardiography were performed. Blood tests, including complete blood count, albumin, alanine aminotransferase, alkaline phosphatase, creatinine, urea and total plasma protein, were conducted to confirm healthy status. Immediately after surgery, the uterus and ovaries were macroscopically evaluated and considered exclusion criteria if abnormalities were detected. Pregnant, lactating, or estrus animals were excluded, based on anamnesis, date of last estrus, and ultrasonographic findings.

Patients were randomly allocated by drawing lots into two groups and underwent open OVH with suspensory ligament rupture either in the morning (6–8 h, Group AM—GAM, n = 10) or in the evening (18–20 h, Group PM—GPM, n = 10). Bitches were hospitalized 48 h before surgery and remained hospitalized 48 h for postoperative assessments. Animals were housed in individual metallic cages at 23 °C, had free access to water, received commercial food according to the owner’s instructions, and were taken to an external area three times daily for urination and defecation, under supervision.

**Figure 1:**
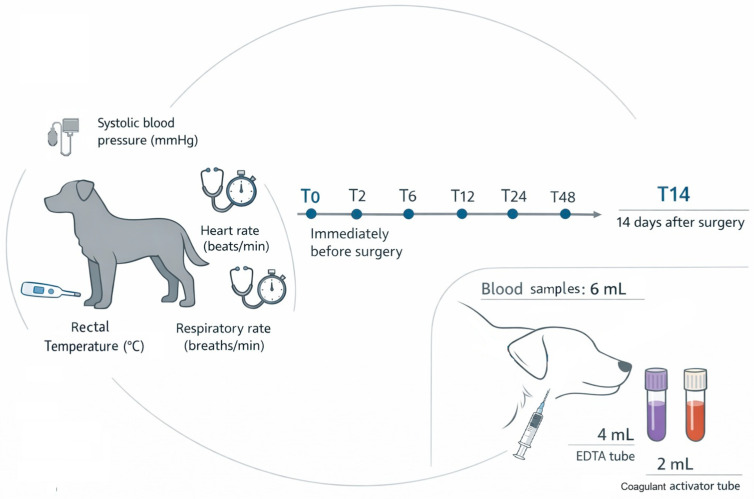
Study design and procedures. Bitches were randomly assigned to one of the groups (GAM, or GPM), with measurements of blood pressure, heart and respiratory rate, temperature, and blood sampling being performed at 7 timepoints. T0 was immediately before surgery (basal), T2-two hours, T6-six hours, T12-12 h, T24-24 h and T48-48 h after surgery, final sample was taken at T14, meaning 14 days after surgery.

After 6 h of food fasting without water restriction, systolic blood pressure (mmHg; ultrasonic Doppler), heart rate (beats/minute; stethoscope and chronometer), respiratory rate (breaths/minute; stethoscope and chronometer) and rectal temperature (°C; digital thermometer) were recorded. Blood samples (6 mL) were collected via jugular venipuncture at T0 (immediately before surgery), and at 2 (T2), 6 (T6), 12 (T12), 24 (T24), 48 (T48) hours and 14 days (T14) postoperatively; 4 mL were placed in EDTA tubes and 2 mL in clot-activator tubes with (Figure 1). All procedures were performed by the same team.

### 2.2. Anesthetic and Surgical Procedures

All procedures were performed by the same trained team, consisting of an experienced surgeon, one assistant, one anesthetist and one nurse, maintaining strict standardization of anesthetic and surgical protocols.

Methadone was administered (0.3 mg/kg IM) (MYTedon, Cristalia, São Paulo, Brazil). After 15 min the abdomen was clipped; cephalic venous access establish, and Ringer’s Lactate solution (5 mL/kg/h IV) was administered by infusion pump (Samtronic–ST 1000, São Paulo, Brazil) until extubation. Sodium ampicillin (20 mg/kg IV) (Ampicilina Sódica, Laboratório Teuto Brasileiro S.A., Anápolis, GO, Brazil) was administered and anesthesia was induced with propofol (6 mg/kg IV-1 mg/kg/10 seg) (Fresofol, Fresenius Kabi, São Paulo, Brazil). After intubation, anesthesia was maintained was with isoflurane (Isoforine, Cristalia, São Paulo, Brazil) vaporized in 100% oxygen using a calibrated vaporizer (Fuji Maximus, Takaoka, Tokyo, Japan) in a closed circuit with partial rebreathing.

An epidural block was performed with patient in sternal recumbency at L7-S1, using lidocaine (0.24 mL/kg) (XYLestesin, Cristalia, São Paulo, Brazil) and morphine (0.1 mg/kg) (Dimorf, Cristalia, São Paulo, Brazil). Heart rate and respiratory rate, electrocardiography and pulse oximetry were monitored using a multiparametric monitor (MR1200VET, RZ Vet, São Paulo, Brazil); systolic blood pressures were accessed by ultrasonic Doppler. After abdominal access, the surgeon infiltrated lidocaine (2 mg/kg) (XYLestesin, Cristalia, São Paulo, Brazil) into the ovarian suspensory ligament, before manipulation. Fentanyl citrate (2.5 µg/kg IV) (Janssen-Cilag Farmacêutica LTDA, São Paulo, Brazil) was administered if a 20% increase in baseline heart rate, respiratory rates, systolic blood pressure, or temperature was observed during surgery.

Procedures were standardized to ensure comparable surgical stimulus between groups; the same team, similar maneuvers and operative time were maintained, totaling approximately 25 min (Supplementary Material Table S1). OVH was performed via ventral midline approach using a scalpel, encompassing one-third of the distance between umbilicus and pubis. After entering the abdominal cavity, the incision was extended with Metzenbaum scissors (EDLO, Canoas, RS, Brazil), followed by identification of the right ovary. Local anesthetic infiltration was performed, followed by rupture of the suspensory ligament [1] and bipolar coagulation. Hemostasis of the uterine horn was performed similarly, including the positioning of the omentum over the uterine body. Muscular closure was performed in a simple continuous pattern using polyglactin 910; the subcutaneous tissue was closed in a continuous pattern with the same suture material, followed by intradermal closure with fine nylon.

Immediately after removal, the uterus, ovaries and uterine tubes were macroscopically evaluated. If alterations were detected, samples were fixed in 10% formalin for histopathological evaluation examination; animals were included only if no abnormalities were identified. Postoperative care included methadone (0.25 mg/kg SC/TID/48 h), local chlorhexidine application (BID), and protective clothes. Pain assessment [21] was performed every 2 h up to 12 h postoperative; if a score was reached fentanyl sulfate (2.5 µg/kg SC) was administered.

### 2.3. Postoperative Evaluation

Clinical evaluation, blood sampling and postoperative procedures were performed by the same researchers. Rectal temperature, heart rate, respiratory rate and systolic blood pressure were evaluated at T0, T2, T6, T12, T24, T48 and T14.

Serum cortisol was evaluated at T0, T2, T6, T12, T24 and T48. For this purpose, 2 mL of whole blood were collected in clot activator tubes, centrifuged at 3500 RPM/10 min and frozen at −80 °C; samples were analyzed simultaneously using chemiluminescence assay [22].

A volume of 4 mL of total blood was collected at T0, T6, T12, T24, T48 and T14 in EDTA tubes for leukogram and oxidative metabolism analyses. The leukogram was performed [23] and the results were interpreted previously described [11]. Differential leucocyte counts were performed in duplicate on stained slides (Diff-Quick method), using light microscopy (1000×) (Olympus^®^ CX21, Tokyo, Japan); including segmented neutrophils, lymphocytes, monocytes and eosinophils [23]; data evaluation was performed [11]. Oxidative stress biomarkers were evaluated at T0, T6, T12, T24, T48 and T14. Protein thiols (P-SH) were measured in plasma and nonprotein thiols (NP-SH) in erythrocyte [24,25]. Vitamin C concentration was estimated in plasma [25,26]. FRAP was determined based on the ability of the sample to reduce Fe^3+^ to Fe^2+^; and the absorbance was measured at (700 nm) [27]. MDA was indirectly evaluated in erythrocyte and plasma using the TBARS method [28], with readings at 532 nm (BIO2000, Bioplus, Seongnam-si, Republic of Korea).

### 2.4. Statistical Analysis

Data normality was assessed using Shapiro–Wilk and homogeneity of variances using Levene’s test. For normally distributed data with homogeneous variances, comparisons between groups were performed using the unpaired test. When variances were heterogeneous, Welch’s correction was applied. For non-normally distributed data, Mann–Whitney U test was used. Within-group comparisons over time were performed using ANOVA followed by Tukey’s post hoc test for normally distributed data, and the Kruskal–Wallis test followed by the Dwass–Steel–Critchlow–Fligner post hoc test for non-normal data. General Linear Models (GLM) were applied when appropriate. Statistical analyses were performed using Jamovi software version 2.3 [29], adopting a significance level of 5% (*p* < 0.05).

## 3. Results

The bitches included in this study comprised different breeds and aged 12–54 months (GAM = 22.1 ± 10.42, GPM 34.1 ± 13.08 months; *p* = 0.043), with body weights ranging from 10.4 to 20.5 kg (GAM = 15.9 ± 3.2, GPM = 18.2 ± 2.93 kg; *p* = 0.114). No patient presented complications during anesthesia, surgery or wound healing; skin sutures were removed after 14 days. No intraoperative or postoperative analgesic rescue was required. No differences between groups were observed regarding postoperative pain scores.

No differences between groups were observed in time to first solid food intake (GAM = 159 ± 48.7 min, GPM = 182 ± 81.9 min; *p* = 0.48) or first defecation (GAM = 394 ± 204.8 min, GPM = 354 ± 367.4 min; *p* = 0.80). Time to first urination differ between groups, occurring earlier in GAM (GAM = 286 ± 143.6 min, GPM = 550 ± 337.2 min; *p* = 0.04).

No differences were observed between groups or over the time for systolic blood pressure (*p* > 0.05) (Supplementary Material Table S2). Heart rate (Supplementary Material Table S3) differed between groups at 14 days, with higher values in GPM (GAM = 111.6 ± 29.15 bpm, GPM = 158 ± 23.81 bpm; *p* = 0.003). Over the time, GAM showed a reduction at 48 h followed by an increase at T14 (*p* = 0.009). In GPM, heart rate decreased at 24 h, returned to baseline at 48 h, and increased above baseline at 14 days (*p* < 0.001). Respiratory rate (Table 1) was higher in GPM at baseline (GAM = 33.8 ± 12.35 bpm, GPM = 53.8 ± 23.5 bpm; *p* = 0.038); at 2 h (GAM = 31.5 ± 7.11 bpm, GPM = 42 ± 13.52 bpm; *p* = 0.049); at 48 h (GAM = 37.7 ± 25.39, GPM = 68 ± 30.4 bpm; *p* = 0.03) and at 14 days (GAM = 40.4 ± 17.02, GPM = 78.9 ± 47.15 bpm; *p* = 0.035). No significant changes over the time were observed within either group. Rectal temperature (Supplementary Material Table S4) was higher in GPM at 12 h (GAM = 37.4 ± 0.3 °C, GPM = 37.9 ± 0.5 °C; *p* = 0.046). Over time, GAM showed a decrease at 2 h returning to baseline at 6 h (*p* < 0.001). GPM exhibited a similar temporal pattern (*p* = 0.001).

**Figure 2:**
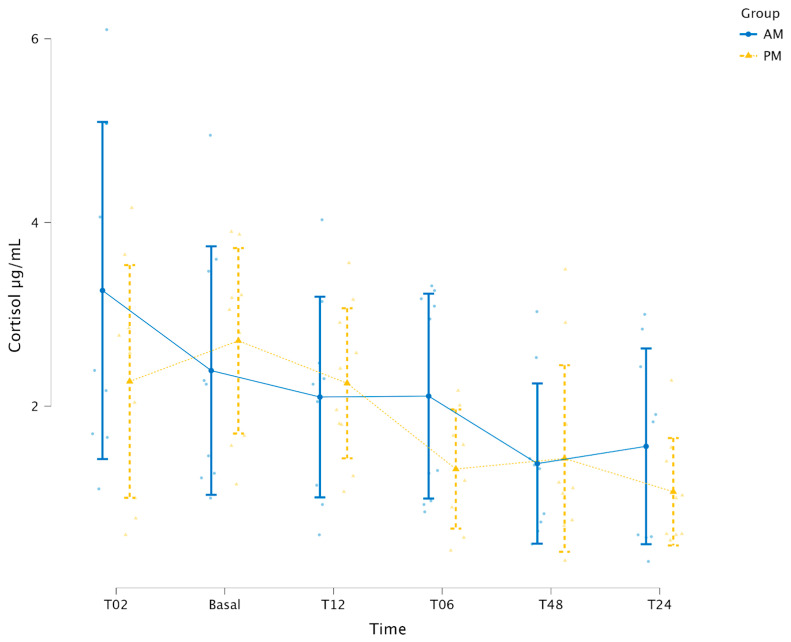
Mean and standard deviation of serum cortisol (μg/dL) between groups and along the time of evaluation in bitches undergoing ovariohysterectomy at the morning (GAM—blue line) or at night (GPM—yellow line).

To evaluate whether cortisol secretion was modulated by the time of surgery, a General Linear Model (GLM) was applied with group (GAM vs. GPM) and timepoint as factors. No group effect was detected (*p* = 0.170), whereas a significant time effect was observed (*p* < 0.001). No significant Group vs. Timepoint interaction was found (*p* = 0.366) (Figure 2).

Regarding leukocytes (Table 2), a difference between groups was observed at 14 days, with higher counts in GAM (*p* = 0.03). Over time, both groups showed increased leukocyte counts at 6 h, peaking at 12 h and returning to baseline at 24 h (*p* < 0.001). Segmented neutrophils did not differ between groups (*p* > 0.05). Over time, GAM showed an increase at 6 h, peaking at 12 h and returning to baseline at 48 h (*p* < 0.001); GPM presented a similar pattern (*p* < 0.001). Lymphocytes and eosinophils (Supplementary Material Tables S5 and S6) did not differ between groups or over time (*p* > 0.05). Monocytes counts differ between groups at 14 days, with higher values in GAM (*p* = 0.008). Over time, GPM peaked at 12 h and returned to baseline at 24 h (*p* < 0.001).

Regarding antioxidants parameters, vitamin C (Table 3) did not differ between groups. Over time, GAM showed a reduction at 24 h, that persisted at 14 days (*p* < 0.001), GPM showed no significant changes over time (*p* > 0.05).

FRAP (Table 4) did not differ between groups (*p* > 0.05). Over time, GAM decreased at 6 h, increasing at 12 h, remained stable at 24 h, increased again at 48 h and returned to baseline at 14 days (*p* = 0.007). GPM increased at 12 h, remained stable at 24 h, increased again at 48 h, and returned to baseline at 14 days (*p* = 0.007).

Protein thiols (Table 5) did not differ between groups (*p* > 0.05). Over time, GAM decreased at 24 h, further at 48 h and returned to baseline at 14 days (*p* < 0.001); GPM presented reduction at 12 h, returned to the basal values at 24 h, decrease again at 48 h, and reached the lowest values at 14 days (*p* < 0.001).

Non-protein thiols (Table 6) a differed between groups at 48 h (*p* = 0.005), with higher values at GPM. Over time, GPM increased at 48 h (*p* = 0.001) and returned to baseline at 14 days, whereas GAM showed no significant changes (*p* > 0.05).

Plasma TBARS (Table 7) differed between groups at 24 h (*p* = 0.01), with higher values in GPM. Over time, GAM increased at 24 h, returned to baseline at 48 h, and showed a further reduction at 14 days (*p* = 0.004). In GPM, values decreased at 12 h, peaked at 24 h, decreased at 48 h and decreased again at 14 days. Erythrocyte TBARS (Supplementary Material Table S7) levels did not differ between groups (*p* > 0.05) or over time (*p* > 0.05).

## 4. Discussion

To minimize stress, patients were hospitalized prior to surgery, allowing acclimatization reducing interference from external stressors on physiological parameters. This management is important, as cortisol levels and oxidative stress makers may increase in animals hospitalized before surgery [30]. Additionally, both groups exhibited increased heart rate at 14 days, when the patients returned to the hospital for evaluation and suture removal. Similar findings have been reported in dogs examined by a veterinarian in the absence of the owner [31], with increases in heart rate and systolic blood pressure attributed to acute stress, as also observed in this study.

This study employed an integrated assessment, including physical and laboratory examination, based on the premise that no single test is capable of fully evaluating the surgical stress response [4,7]. The authors do not consider that the age difference between groups influenced the physiological results, according the Canine Life Stage Guidelines [32], which classify dogs as young adults after one year of age, with physical and social maturation generally completed between three and four years.

Regarding physical parameters, no differences in systolic blood pressure were observed between groups or over time (*p* > 0.05), consistent with previous comparable OVH techniques in bitches [8,33]. The increase in heart rate at 14 days, was attributed to acute stress associated with hospital return [31], as discussed above.

Respiratory rate differed between groups, with higher values in GPM at baseline (*p* = 0.038), 2 h (*p* = 0.049), 48 h (*p* = 0.030) and 14 days (*p* = 0.035). The higher baseline and 48 h values in GPM may be attributed to the time of the day of evaluation (18–20 h), when animals are fully awake, whereas GAM baseline measurements were obtained during early morning hours (6:00–8:00 h), when patients were more likely to be drowsy. These suggest an influence of sleep-wake state rather than a difference in surgical stress response [34]. According to the same authors, respiratory frequency is lower during light sleep or transitional states (drowsiness), as observed in GAM; whereas higher values are reported during wakefulness or rapid eye movement sleep (REM), as seen in GPM [34]. At 2 h postoperatively, higher respiratory rates were observed in GPM. At this time point, sleep-wake interference is less likely [34]; therefore, alteration may cautiously be associated with the surgical stress response, considering the sensibilization of nociceptive pathways to the brainstem, thalamus, and hypothalamus, which stimulate the respiratory centers [4].

Both groups exhibited a reduced rectal temperature at 2 h postoperatively (GAM *p* < 0.001 and GPM *p* = 0.001), returning to baseline by 6 h, GPM values were higher than GAM (*p* = 0.046). Although circadian rhythm may influence body temperature, with higher values during the light phase and lower during dark phase [34,35], this pattern was not clearly observed. The transient reduction was attributed to postoperative hypothermia, which may be more pronounced after open OVH compared to video surgery [8].

No differences were observed between groups in time to first food intake or defecation (*p* > 0.05), although reduced gastrointestinal motility was recognized after celiotomy due to increased plasma catecholamines [8]. However, GAM showed a shorter time to first urination (*p* = 0.04). Surgical procedures that induce hormonal alterations involved in fluid balance regulation, arginine and vasopressin are released by the hypophysis in response to hypovolemia, anesthesia, or pain promoting water retention and urine concentration [4,36]. Therefore, the longer time to first urination in GPM may suggest a slightly greater homeostatic disturbance in animals operated at night.

No difference in cortisol levels were observed between groups (*p* > 0.05). Temporal variation was detected in both groups (*p* < 0.001), consistent with circadian rhythm patterns [37]. These findings align with a study evaluating salivary and serum cortisol every 3 h, over 48 h, in healthy dogs under controlled conditions, which reports lower cortisol concentrations during the dark phase [37]. In the present study, both groups showed lower values during the dark phase compared with the light phase, suggesting that the timing of OVH did not alter the cortisol secretion profile. Furthermore, no increase in cortisol levels was observed 2 h after surgery, similar to findings in bitches undergoing 30 min OVH evaluated 3 h later [7]. Conversely, other studies have reported increased cortisol levels after OVH [38]. The absence of elevation in this study may be attributed to the efficacy of the analgesic protocol since central nervous system stimulation by pain can promote adrenocorticotropic hormone release and subsequent, adrenal secretion of cortisol, corticosterone and aldosterone [4,39].

Leukocytosis was observed in both groups at 6 h, returning to baseline at 24 h (*p* < 0.001). Neutrophilia was observed in both groups at 6 h (*p* < 0.001) returning to baseline at 48 h. Neutrophilia is typical of acute inflammation and reflects redistribution from the storage pool [11], consistent with findings from other OVH studies using approaches with bipolar hemostasis [8,33,40].

Monocyte counts increased at 12 h in GPM, (*p* < 0.001), whereas no temporal changes were observed in GAM. Monocytosis may be triggered by corticosteroids or pro-inflammatory cytokines [11] and may reflect a stress-related response [4]. Similar findings have been reported in open OVH studies [40]. Although GPM showed reduced monocyte counts at T14, this was not considered a monocytopenia, as monocyte numbers are typically low in peripheral blood [11].

No differences were observed in lymphocyte and eosinophil counts (*p* > 0.05). Lymphopenia and eosinopenia may occur after stress due to redistribution and reduced bone marrow release [11]. The absence of these alterations supports the hypothesis of a relatively mild homeostatic disturbance, consistent with previous reports evaluating hematological and biochemical changes after OVH [40].

Regarding the oxidative stress markers, P-SH did not differ between groups (*p* > 0.05), but both decreased at 48 h, and remained reduced at 14 days (*p* < 0.001). ROS target unsaturated lipids and intracellular thiols; lipid peroxidation generate metabolites that react rapidly with the thiols group [14]. Additionally, enzymatic antioxidant systems consume thiols as reducing agents [14]. These findings are consistent with previous studies evaluating open OVH in bitches at 14 and 30 days after surgery [41], which report persistent reduction beyond the baseline.

NP-SH increased in GPM at 48 h and returned to baseline levels at 14 days. This marker reflects non-protein, non-enzymatic antioxidant, such as glutathione (GSH) [42]. Although, consumption of these compounds is generally expected after surgery, the increase observed contrasts with previous findings in bitches subjected to OVH with suspensory ligament rupture, in which no alterations were detected [33]. Another study measuring GSH levels in bitches 30 and 180 days after flank OVH reported a chronic reduction in these antioxidant levels [15]. Therefore, the elevation of thiol levels at 48 h in GPM may indicate a compensatory increase in the endogenous GSH, as previously described in humans [43].

Vitamin C did not differ between groups; however, GAM showed a reduction at 24 h, partial recovery at 48 h and a decrease at 14 days (*p* < 0.001). As vitamin C inhibits lipid peroxidation, its reduction may reflect antioxidant consumption [44]. Considering the higher TBARS levels in GPM, the vitamin C reduction observed in GAM may have been sufficient to prevent lipidic peroxidation at 24 h in this group.

FRAP did not differ between groups; however, over time, both GAM and GPM showed reduction at 24 h and returned to baseline at 48 h (*p* = 0.007). This biomarker reflects antioxidant capacity through a reduction in ferric ion [16]. Therefore, the decrease observed at 24 h may indicate antioxidant consumption, given that baseline levels were restored at 48 h. The absence of differences related to the timing OVH is consistent with other studies evaluating FRAP under different conditions; one study compared FRAP in lean and obese dogs [45], and compared open and video assisted OVH [46], both reporting no significant differences in this biomarker.

Regarding TBARS, there was a difference between groups at 24 h, with higher values in GPM (*p* = 0.01). TBARS levels increase as a consequence of lipid peroxidation induced by excessive production of ROS [17], suggesting greater oxidative damage in patients operated in the evening. Over time, TBARS peaked at 24 h in both groups, decreased at 48 h, and returned to baseline at 14 days. These findings differ from another study that reported elevated TBARS, at 14 days and 30 days after OVH [41]. This discrepancy may be associated with differences in anesthetic protocol, as the use of ketamine and xylazine may induce greater oxidative stress compared to inhalatorial anesthesia.

The present findings suggest a greater hemostatic disturbance in patients operated in the evening. Experimental studies in bitches supplemented with melatonin undergoing open OVH demonstrated increased superoxide dismutase, glutathione peroxidase and catalase activity, along with reduced MDA levels [47]. Therefore, the milder disturbance observed in GAM may be associated with preserved endogenous melatonin production, potentially attenuating oxidative surgical stress. In humans surgical procedures are associated with altered circadian melatonin secretion due to fatigue, pain, reduced general welfare, cognitive dysfunction and comorbidity [48], and lower endogenous melatonin levels have been reported during the first postoperative night compared to subsequent nights [49]. Nevertheless, further veterinary studies evaluating perioperative melatonin concentration or supplementation in dogs are required to support this hypothesis.

Although this study was conducted in healthy patients, under standardized conditions, OVH performed at night appeared to induce a slightly more pronounced homeostatic disturbance. This interpretation is supported by longer time to first urination, higher respiratory rates, increased monocyte counts, and greater lipid peroxidation in GPM. Considering that the magnitude of surgical stress depends on the intensity and duration of anesthetic and surgical stimuli [4], a more pronounced effect might be expected in patients of different ages, with comorbidities, infection, inadequate corporal condition, or undergoing longer or more invasive procedures. In such cases, performing elective surgeries in the morning may be preferable Further investigations, including larger sample sizes and additional biomarkers, are necessary to clarify the clinical relevance of the homeostatic alteration observed in this study.

## 5. Conclusions

The OVH in healthy and young bitches conducted at night induced a small homeostasis alteration, which was re-established 14 days after surgery, considering the specific anesthetic and surgery protocols used. The clinical relevance of these results must be investigated.

## Figures and Tables

**Table 1:** Mean and standard deviation (M ± SD) of the respiratory rate (breath/min) in bitches undergoing ovariohysterectomy in the morning (6–8 h; GAM) or at night (18–20 h; GPM). Columns present the results of each group; lines present the different times of evaluation (*p* < 0.05).

Time	GAM M ± SD	GPM M ± SD	*p* Value *
Basal	33.8 ± 12.35	53.8 ± 23.5	**0.038**
2 h	31.5 ± 7.11	42 ± 13.52	**0.049**
6 h	36.4 ± 21.21	46.2 ± 21.74	0.34
12 h	37.4 ± 18.89	47 ± 20.81	0.33
24 h	44.5 ± 23.38	44.6 ± 15.57	0.99
48 h	37.7 ± 25.39	68 ± 30.4	**0.03**
14 days	40.4 ± 17.02	78.9 ± 47.15	**0.035**
*p* value	0.78	0.056	

* Values in bold indicate statistical significance at *p* < 0.05.

**Table 2:** Mean and standard deviation (M ± SD) of total leukocytes (cells/μL), segmented neutrophils (cells/μL) and monocytes (cells/μL) of bitches undergoing ovariohysterectomy in the morning (6–8 h; GAM) or at night (18–20 h; GPM). Columns present the results of each group; lines present the different times of evaluation. Lowercase letters indicate statistical differences (*p* < 0.05).

Total Leukocytes			
Time	GAM M ± SD	GPM M ± SD	*p* Value *
Basal	14,456 ± 3558 a	13,513 ± 2954 a	0.56
6 h	21,656 ± 3787 bc	22,122 ± 5110 b	0.82
12 h	23,733 ± 4810 bc	22,889 ± 5621 b	0.73
24 h	18,420 ± 4438 abd	19,356 ± 5567 abc	0.68
48 h	14,811 ± 2684 ad	15,667± 3456 acd	0.56
14 days	15,283 ± 1546 ad	12,229 ± 2699 ad	**0.03**
*p* value *	**<0.001**	**<0.001**	
Neutrophils			
Time	GAM M ± SD	GPM M ± SD	*p* Value
Basal	7551 ± 2421 a	8743± 3143 ac	0.35
6 h	15,961 ± 3744 bc	17,136 ± 4227 b	0.52
12 h	18,434 ± 5613 bc	19,017 ± 5860 b	0.82
24 h	12,606 ± 4507 bd	14,772 ± 5694 bc	0.35
48 h	8249 ± 1792 ad	9300 ± 2531 acd	0.29
14 days	8326 ± 708 ad	7541 ± 2152 ad	0.41
*p* value *	**<0.001**	**<0.001**	
Monocytes			
Time	GAM M ± SD	GPM M ± SD	*p* Value *
Basal	1071 ± 494	851 ± 461 ac	0.34
6 h	1615 ± 664	1118 ± 426 abc	0.07
12 h	1446 ± 686	1666 ± 546 bc	0.45
24 h	1156 ± 403	1560 ± 753 abcd	0.20
48 h	1283 ± 495	1231 ± 422 abcde	0.80
14 days	1104 ± 325	572 ± 232 abe	**0.008**
*p* value *	0.24	**<0.001**	

* Values in bold indicate statistical significance at *p* < 0.05.

**Table 3:** Mean and standard deviation (M ± SD) of vitamin C (ug/mL plasma) of bitches undergoing ovariohysterectomy in the morning (6–8 h; GAM) or at night (18–20 h; GPM). Columns present the results of each group and the lines the different times of evaluation. Lowercase letters indicate statistical differences to GAM and GPM (*p* < 0.05).

Time	GAM M ± SD	GPM M ± SD	*p* Value *
Basal	11.77 ± 1.13 a	12.44 ± 2.05	0.37
6 h	11.34 ± 1.44 a	12.15 ± 1.48	0.23
12 h	10.50 ± 1.63 ab	10.69 ± 1.78	0.80
24 h	9.44 ± 0.90 b	9.81 ± 1.38	0.48
48 h	10.46 ± 1.71 ab	9.97 ± 1.47	0.50
14 days	9.51 ± 1.30 b	9.54 ± 1.49	0.95
*p* value *	**<0.001**	0.058	

* Values in bold indicate statistical significance at *p* < 0.05.

**Table 4:** Mean and standard deviation (M ± SD) of ferric reducing ability of plasma (FRAP) (μg/mL plasma) of bitches undergoing ovariohysterectomy in the morning (6–8 h; GAM) or at night (18–20 h; GPM). Columns present the results of each group and lines the different times of evaluation. Lowercase letters indicate statistical differences (*p* < 0.05).

Time	GAM M ± SD	GPM M ± SD	*p* Value *
Basal	458 ± 77 abc	456 ± 92.1 abc	0.96
6 h	380 ± 83.7 ab	383 ± 59.6 abc	0.92
12 h	412 ± 44.3 abc	393 ± 47.7 ab	0.36
24 h	398 ± 82 abc	382 ± 44.5 ab	0.59
48 h	483 ± 43.8 ac	455 ± 32.7 ac	0.12
14 days	466 ± 76.7 abc	418 ± 26.8 abc	**0.07**
*p* value *	**0.007**	**0.007**	

* Values in bold indicate statistical significance at *p* < 0.05.

**Table 5:** Mean and standard deviation (M ± SD) of protein thiols (μmol/mL plasma) of bitches undergoing ovariohysterectomy in the morning (6–8 h; GAM) or at night (18–20 h; GPM). Columns present the results of each group and the lines the different times of evaluation. Lowercase letters indicate statistical differences to GAM and GPM (*p* < 0.05).

Time	GAM M ± SD	GPM M ± SD	*p* Value *
Basal	9.37 ± 2.99 ab	12.15 ± 3.53 a	0.08
6 h	12.82 ± 1.24 ab	11.82 ± 3.34 a	0.38
12 h	11.57 ± 3.72 ab	10.37 ± 3.44 ab	0.46
24 h	10.65 ± 3.28 abc	10.93 ± 3.45 a	0.86
48 h	5.09 ± 2.75 ad	6.72 ± 2.28 bc	0.16
14 days	7.08 ± 2.62 abcd	5.64 ± 2.73 c	0.24
*p* value *	**<0.001**	**<0.001**	

* Values in bold indicate statistical significance at *p* < 0.05.

**Table 6:** Mean and standard deviation (M ± SD) of non-protein thiols (μmol/mL erythrocytes) of bitches undergoing ovariohysterectomy in the morning (6–8 h; GAM) or at night (18–20 h; GPM). Columns present the results of each group and the lines the different times of evaluation. Lowercase letters indicate statistical differences to GAM and GPM (*p* < 0.05).

Time	GAM M ± SD	GPM M ± SD	*p* Value *
Basal	9.29 ± 4.06	9.06 ± 3.94 a	0.89
6 h	8.70 ± 1.37	9.20 ± 3.24 a	0.67
12 h	10.38 ± 3.03	9.60 ± 4.74 ab	0.66
24 h	9.82 ± 3.58	10.03 ± 2.51 ab	0.87
48 h	9.90 ± 2.68	13.86 ± 2.69 b	**0.005**
14 days	11.86 ± 3.60	11.77 ± 2.71 ab	0.948
*p* value *	0.46	**0.001**	

* Values in bold indicate statistical significance at *p* < 0.05.

**Table 7:** Mean and standard deviation (M ± SD) of the thiobarbituric acid reactive substances in plasma (TBARS) (nmol MDA/mL plasma) of bitches undergoing ovariohysterectomy in the morning (GAM) or at night (GPM). Columns present the results of each group and the lines the different times of evaluation. Lowercase letters indicate statistical differences to GAM and GPM (*p* < 0.05).

Time	GAM M ± SD	GPM M ± SD	*p* Value *
Basal	31.4 ± 5.96 a	30.3 ± 6.21 a	0.68
6 h	22.7 ± 12.75 a	24.8 ± 11.16 a	0.70
12 h	31.0 ± 7.26 a	26.4 ± 9.17 ab	0.23
24 h	31.3 ± 6.20 ab	39.3 ± 6.81 ac	**0.01**
48 h	27.5 ± 3.57 a	32.1 ± 6.89 a	0.07
14 days	21.7 ± 5.05 ac	24.6 ± 4.98 ab	0.21
*p* value *	**0.004**	**<0.001**	

* Values in bold indicate statistical significance at *p* < 0.05.

## Data Availability

All of the data is contained within this paper.

## Supplementary Materials

The following supporting information can be downloaded at: https://www.mdpi.com/article/10.3390/ani16050795/s1, Table S1: Patronization of the surgical time and procedures in bitches undergoing ovariohysterectomy in the morning (GAM) or at night (GPM). Clinical and Laboratorial data: Table S2: Mean and standard deviation (M ± SD) of the PAS (mmHg) in bitches undergoing ovariohysterectomy in the morning (GAM) or at night (GPM); Table S3: Mean and standard deviation (M ± SD) of the heart rate (bpm) in bitches undergoing ovariohysterectomy in the morning (GAM) or at night (GPM); Table S4: Mean and standard deviation (M ± SD) of the rectal temperature (°C) in bitches undergoing ovariohysterectomy in the morning (GAM) or at night (GPM); Table S5: Mean and standard deviation (M ± SD) of the Lymphocytes (cells/μL) of bitches undergoing ovariohysterectomy in the morning (GAM) or at night (GPM); Table S6: Mean and standard deviation (M ± SD) of the Eosinophil (cells/μL) of bitches undergoing ovariohysterectomy in the morning (GAM) or at night (GPM); Table S7: Mean and standard deviation (M ± SD) of the erythrocyte substances reactives of the thiobarbituric acid (TBARS) (nmol MDA/mL erythrocytes) of bitches undergoing ovariohysterectomy in the morning (GAM) or at night (GPM).
