# Supplementary material for: Influence of Circadian Rhythm on the Surgical Stress Response in Bitches Undergoing Elective Ovariohysterectomy
Source: Animals (Basel). 2026 Mar 4;16(5):795. doi: 10.3390/ani16050795 (PMC12984636; doi:10.3390/ani16050795)
Supplement: Supplementary file 1 [file animals-16-00795-s001.zip › animals-4149184-supplementary.pdf]

Supplementary file: Influence of Circadian Rhythm on the Surgical Stress Response in Bitches Undergoing Elective Ovariohysterectomy

Table S1. Padronization of the surgical time and procedures in bitches undergoing ovariohysterectomy in the morning (GAM) or at night (GPM).

**Pre-operative**	
**Procedure**	**Duration (minutes)**
Application of preanesthetic medication	15
Trichotomy	10
Cephalic vein access and fluid therapy	5
Induction/intubation	4
Epidural anesthesia	10
Animal positioning and antisepsis	3
**Total time**	**50**
**Surgery**	
**Procedure**	**Duration (minutes)**
Positioning and securing of surgical drapes	3
Skin, subcutaneous and muscular incision	2
Localization of the right ovary, administration of lidocaine, rupture of the ligament, and cauterization of the arteriovenous complex	4
Localization of the left ovary, administration of lidocaine, rupture of the ligament, and cauterization of the arteriovenous complex	4
Localization of the uterine body and cauterization	2
Examination of the abdominal cavity and omentalization	1
Muscle layer closure	4
Closure of the subcutaneous	2
Skin closure	3
**Total time**	**25**

CLINICAL AND LABORATORIAL DATA

Table S2. Mean and standard deviation (M ± SD) of the PAS (mmHg) in bitches undergoing ovariohysterectomy in the morning (GAM) or at night (GPM). Columns present the results of each group; lines present the different times of evaluation.

**Tempo**	**GAM**<br>**M ± SD**	**GPM**<br>**M ± SD**	**P *value***
**Basal**	150 ± 27.3	159 ± 21.0	0.47
**2h**	143 ± 19.5	160 ± 27.9	0.14
**6h**	163 ± 29.4	171 ± 36.2	0.62
**12h**	155 ± 23.4	161 ± 22.2	0.55
**24h**	152 ± 17.8	151 ± 23.2	0.96
**48h**	152 ± 23.3	156 ± 15.2	0.67
**14 dias**	168 ± 31.2	167 ± 20.9	0.92
**P *value***	0.40	0.71	

Table S3. Mean and standard deviation (M ± SD) of the heart rate (bpm) in bitches undergoing ovariohysterectomy in the morning (GAM) or at night (GPM). Columns present the results of each group; lines present the different times of evaluation. Lowercase letters indicate statistical differences ($p < 0.05$).

**Tempo**	**GAM**<br>**M ± SD**	**GPM**<br>**M ± SD**	**P *value***
**Basal**	106 ± 18.50abc	106.4 ± 20.41a	0.96
**2h**	89.8 ± 12.18abc	83.1 ± 15.85ab	0.33
**6h**	90.8 ± 9.81abc	92.8 ± 16.42ab	0.74
**12h**	91.6 ± 12.61abc	96 ± 9.38ab	0.36
**24h**	90.2 ±10.02abc	82.7 ± 11.83b	0.16
**48h**	86.2 ± 13.28ab	85.6 ± 15.11ab	0.92
**14 dias**	111.6 ± 29.15ac	158 ± 23.81c	**0.003**
**P *value***	**0.009**	**<0.001**	

Table S4. Mean and standard deviation (M ± SD) of the rectal temperature (ºC) in bitches undergoing ovariohysterectomy in the morning (GAM) or at night (GPM). Columns present the results of each

group; lines present the different times of evaluation. Lowercase letters indicate statistical differences ($p < 0.05$).

Tempo	GAM<br>M ± SD	GPM<br>M ± SD	P *value*
**Basal**	38.3 ± 0.41a	38.5 ± 0.33ac	0.14
**2h**	37.4 ± 0.34b	37.9 ± 0.55bc	**0.046**
**6h**	38.1 ± 0.47ab	38.3 ± 0.32abc	0.26
**12h**	38.2 ± 0.37a	38.0 ± 0.50abc	0.35
**24h**	38 ± 0.37a	38 ± 0.38abc	0.90
**48h**	37.8 ± 0.38ab	37.9 ± 0.39ab	0.69
**14 dias**	38.5 ± 0.52a	38.6 ± 0.47ac	0.65
**P *value***	**<0.001**	**0.001**	

Table S5. Mean and standard deviation (M ± SD) of the Lymphocytes (cells/μL) of bitches undergoing ovariohysterectomy in the morning (GAM) or at night (GPM). Columns present the results of each group and the lines the different times of evaluation.

Tempo	GAM<br>M ± SD	GPM<br>M ± SD	P *value*
**Basal**	2756 ± 933	2043 ± 950	0.12
**6h**	2251 ± 700	1785 ± 673	0.18
**12h**	2296 ± 840	1893 ± 426	0.27
**24h**	2772 ± 1156	2489 ± 989	0.59
**48h**	2850 ± 1163	2672 ± 936	0.71
**14 dias**	2894 ± 764	2387 ± 759	0.27
**P *value***	0.59	0.19	

Table S6. Mean and standard deviation (M ± SD) of the Eosinophil (cells/μL) of bitches undergoing ovariohysterectomy in the morning (GAM) or at night (GPM). Columns present the results of each group and the lines the different times of evaluation..

Tempo	GAM<br>M ± SD	GPM<br>M ± SD	P *value*

**Basal**	1459 ± 774	1465 ± 671	0.96
**6h**	1041 ± 843	1128 ± 748	0.81
**12h**	698± 841	1498 ± 1107	0.14
**24h**	1115 ± 1083	1680 ± 1026	0.25
**48h**	988 ± 439	1612 ± 819	0.08
**14 dias**	1837 ± 1228	1460 ± 1099	0.57
**P *value***	0.06	0.74	

Table S7. Mean and standard deviation (M ± SD) of the erythrocyte substances reactives of the thiobarbituric acid (TBARS) (nmol MDA/mL erythrocytes) of bitches undergoing ovariohysterectomy in the morning (GAM) or at night (GPM). Columns present the results of each group and the lines the different times of evaluation.

**Tempo**	**GAM M ± SD**	**GPM M ± SD**	**P *value*****
**Basal**	18.6 ± 4.56	17.7 ± 3.64	0.65
**6h**	20.0 ± 4.58	17.4 ± 2.83	0.14
**12h**	18.8± 3.21	19.5 ± 3.54	0.62
**24h**	17.7 ± 3.01	20.1 ± 4.32	0.16
**48h**	17.4 ± 2.97	19.9 ± 3.80	0.12
**14 dias**	17.7 ± 4.41	19.5 ± 2.29	0.28
**P *value***	0.73	0.38	
